# Case Report: Multiple types of arrhythmias in a late-confirmed Danon disease

**DOI:** 10.3389/fcvm.2024.1369680

**Published:** 2024-03-28

**Authors:** Nan Wang, Yu Cao, Jie Wang, Qing Zhang

**Affiliations:** Department of Cardiology, West China Hospital, Sichuan University, Chengdu, China

**Keywords:** Danon disease, arrhythmia, Wolff–Parkinson–White (WPW) syndrome, *LAMP2* variant, case report

## Abstract

**Introduction:**

Danon disease is an X-linked disorder caused by pathogenic variants in lysosome-associated membrane protein 2 (*LAMP2*) gene, typically characterized by the triad of hypertrophic cardiomyopathy, myopathy, and intellectual disability. However, many patients may not present the typical presentation, especially in the early stage. Electrocardiogram (ECG) abnormalities can be found in almost all patients, with Wolff–Parkinson–White (WPW) syndrome being the most common. We reported the case of a 51-year-old woman who experienced multiple types of arrhythmias over three decades and was diagnosed with Danon disease late by genetic testing.

**Case summary:**

A 51-year-old woman with a 36-year history of intermittent palpitations was admitted due to hemodynamically stable ventricular tachycardia (VT). Her past medical history revealed multiple arrhythmias and ECG abnormalities in her 30s and 40s, including WPW syndrome with paroxysmal supraventricular tachycardia, paroxysmal atrial flutter, atrial fibrillation, ventricular tachycardia, and complete left bundle branch block. She denied any family history of cardiovascular disease or sudden death. Upon arrival, her vital signs were unremarkable. Cardiovascular magnetic resonance (CMR) imaging revealed left ventricular enlargement and late gadolinium enhancement (LGE) in the anterior, inferior, and lateral walls. Subsequent, whole-exome sequencing (WES) gene testing revealed a pathogenic heterozygous variant in *LAMP2* gene (c.696T>A; p.Cys232Ter), which confirmed the diagnosis of Danon disease.

**Conclusion:**

Genetic testing should be considered in patients who display multiple arrhythmias with LV structural abnormalities of unknown etiology for a possible Danon disease.

## Introduction

Danon disease is a rare X-linked dominant multisystemic disorder characterized by severe cardiac and extracardiac manifestations, including neurologic, skeletal, and ophthalmologic impairments (1). Symptoms related to cardiovascular involvement are among the most prominent and severe clinical manifestations, which often trigger clinical evaluation (1–5). A variety of arrhythmias have been described in patients with Danon disease, including heart block, delta waves, supraventricular arrhythmias (atrial flutter, atrial fibrillation), and ventricular arrhythmias, with the most common type of electrocardiogram (ECG) abnormality being Wolff–Parkinson–White (WPW) syndrome (present in 68% of men and 27% of women) (3, 6, 7). Left ventricular (LV) structural involvement could manifest as hypertrophic or dilated patterns. Because of the X-linked inheritance, male patients with Danon disease are affected earlier and more severely than females (2). For female patients who exhibit arrhythmias without extracardiac manifestations or early cardiac structural changes, such as left ventricular hypertrophy or dilation, the disease is more insidious. This case introduced describes a 51-year-old woman who experienced multiple arrhythmias and WPW syndrome over 30 years without typical cardiac imaging and extracardiac manifestations.

## Case presentation

**Figure 1:**
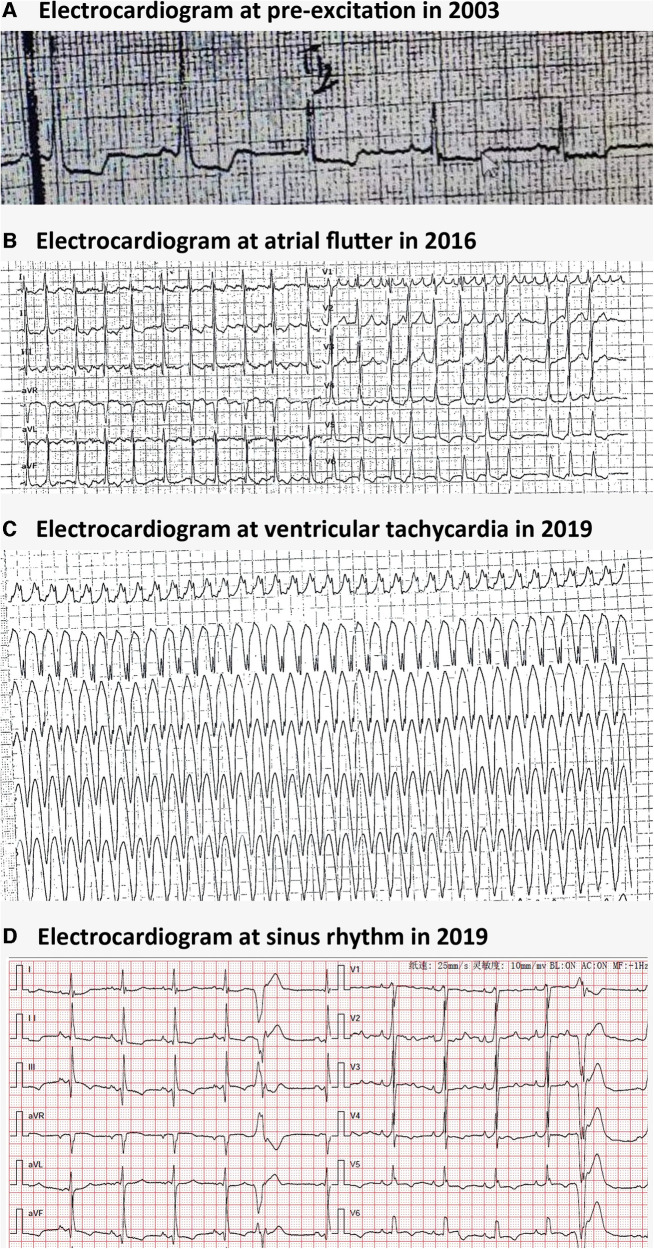
Electrocardiographic findings in Danon disease. (**A**) Electrocardiogram at pre-excitation in 2003. (**B**) Electrocardiogram at atrial flutter in 2016. (**C**) Electrocardiogram at ventricular tachycardia in 2016. (**D**) Electrocardiogram at sinus rhythm with ventricular premature beats and a complete left bundle branch block in 2019.

**Figure 2:**
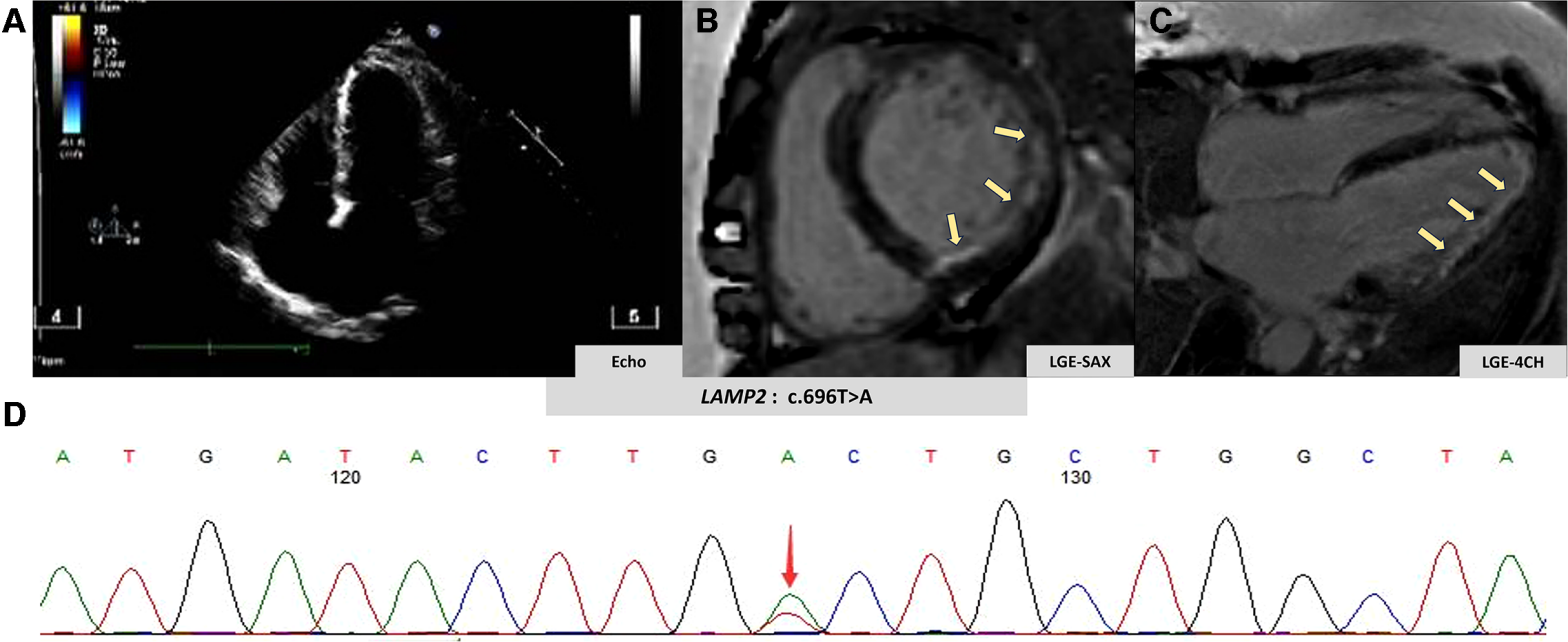
Imaging and genetic findings in Danon disease. (**A**) Patient's echocardiography view. (**B,C**) Patient's late gadolinium enhancement by CMR (yellow arrow). (**D**) Genetic analysis of the clinically affected member.

A 51-year-old woman was admitted to our emergency department due to hemodynamically stable ventricular tachycardia (VT). Her family history was unremarkable for cardiovascular disease, sudden death, or other inherited disorders. Apart from a long history of arrhythmias, she had no special medical or psychosocial record. The patient experienced recurrent paroxysmal palpitations since she was 15 years old. In her 30s, she was diagnosed with Wolff–Parkinson–White (WPW) syndrome (Figure 1A) with paroxysmal supraventricular tachycardia, for which she received her first radiofrequency catheter ablation (RFCA). Echocardiography at that time showed a normal heart, while cardiac troponin levels were not tested. Three years ago, she was admitted with paroxysmal atrial flutter (Figure 1B), during which echocardiography revealed an enlargement of the left atrium (45 mm) but normal left ventricular wall thickness (10 mm), chamber size (51 mm), and ejection fraction (EF) (72%). She was discharged after the RFCA for atrial fibrillation (AF) with a normal troponin-T level. This was her third hospitalization for palpitations. Upon admission, ventricular tachycardia (Figure 1C) was recorded, which was electrically converted to sinus rhythm with a complete left bundle branch block (Figure 1D). Despite no heart murmurs or abnormalities of other systems found in physical examination, a slightly elevated N-terminal pro-B-type natriuretic peptide (568 pg/mL, normal reference value: 0–227 ng/L), markedly increased troponin-T (168.7 ng/L, normal reference value: 0–14 ng/L), and creatine kinase (1,198 IU/L, normal reference value: 19–226 IU/L) were identified. Echocardiography revealed a slightly dilated left ventricle of 55 mm and a slightly decreased EF of 51%. The LA remained enlarged, but the wall thickness was normal (Figure 2A). Cardiovascular magnetic resonance (CMR) imaging was further performed, which displayed endomyocardial late gadolinium enhancement (LGE) in the anterior, inferior, and lateral walls (Figures 2B,C). The following whole-exome sequencing (WES) demonstrated a heterozygous variant in lysosome-associated membrane protein 2 (*LAMP2*) gene (c.696T>A; p.Cys232Ter) (Figure 2D), which is located in coding exon 5 of *LAMP2*. According to ClinVar records, this mutation changes the amino acid from a cysteine to a stop codon, which is expected to result in an absent or disrupted protein product (1). Because of the pathogenic gene mutation with multiple arrhythmias and myocardial involvement, the diagnosis of Danon disease was confirmed. She received the third RFCA, followed by implantable cardioverter defibrillator implantation.

## Discussion

Danon disease, initially described in 1981 by Danon et al., is a rare X-linked dominant multisystemic disorder involving the heart, skeletal muscle, nervous system, retina, and other organs (1, 8). The disease is now considered to result from loss-of-function mutations in *LAMP2* gene, which functions as a lysosomal membrane receptor in chaperone-mediated autophagy (9). The disabled gene leads to the accumulation of glycogen and dysfunction of autophagy, which results in impaired cardiac contractility and conduction abnormalities (10).

For X-dominant inherited disorders such as Danon disease, female patients with heterozygous mutations usually have milder and later clinical manifestations due to the condition of haploinsufficiency (2). This heterogenous penetrance and expressivity in female individuals is thought to be due to skewed X-chromosome inactivation and functional mosaicism of LAMP-2 expression (2). The average age of symptom onset was 12.1 (±6.5) years and 28.1 (±15) years in men and women, respectively (1). The average age of diagnosis, defined as skeletal or cardiac myopathy, cardiac preexcitation, or confirmed Danon disease, was 13.5 (±7.0) years and 31.4 (±15.4) years in men and women, respectively (1). Furthermore, men may be more vulnerable to multisystemic disorders. Symptomatic muscle disease, symptomatic respiratory disease, and learning and cognitive disorders are reported more frequently in men than in women, except for neuropathy, which was reported three times more often in women than in men (1). In this case, the patient presented with isolated cardiovascular disease and was socially well-adjusted. Neither electromyography (EMG) demonstrated any positive results, nor did the patient exhibited any other clinical manifestations of extracardiac impairment. The LV remodeling phenotype also varies between different genders, with male patients typically displaying a concentric LV hypertrophy, whereas female individuals may present with an asymmetric LV hypertrophy, as well as both hypertrophic and dilated forms (11, 12).

Extracardiac manifestations are usually mild to moderate, but cardiac dysfunction plays a life-threatening role in the natural history of Danon disease (2). Arrhythmias are very common in Danon disease, with WPW syndrome being the most common type and perhaps an early marker of the disease (13). In this case, the patient developed WPW syndrome in the early stage and was subsequently readmitted due to other arrhythmias including atrial fibrillation. Coban-Akdemir et al. found an increased burden of rare deleterious variants in genes linked to atrial fibrillation in WPW syndrome (14).

The role of radiofrequency ablation as an effective treatment for arrhythmias may be limited in patients with Danon disease. One of the reasons should be the extensive and accelerated progression of myocardial fibrosis (2). Furthermore, ICD is not as effective for Danon disease as other heart diseases, and sudden cardiac death remains inevitable in patients receiving ICD (15). According to a study of Maron et al., of seven patients with Danon disease who had ICD implanted, five patients died due to failed termination of the fatal tachycardia (16). For most male patients, heart transplantation becomes inevitable in their second and third decades of life (5). For individuals with a loss-of-function mutation in *LAMP2*, gene editing therapy has a promising future, and a clinical trial is underway (ID: NCT03882437) (17).

The diagnosis of Danon disease is usually established in patients with suggestive findings, followed by identifying a hemizygous pathogenic variant in *LAMP2* gene. The rarity of the disease makes it even more difficult in the early stage when typical cardiac and extracardiac manifestations are absent, as observed in this patient (5). It is impractical and not cost-effective to conduct large-scale genetic testing in patients with a single common arrhythmia, such as WPW with supraventricular tachycardia. However, according to the diagnostic algorithm for Danon disease (5), when female patients experience multiple arrhythmias accompanied by abnormal cardiac structure and late gadolinium enhancement on CMR, genetic testing should be considered to confirm or exclude Danon disease.

## Conclusions

Danon disease is difficult to recognize due to its rarity and hidden early manifestations. Genetic testing should be considered for earlier identification in patients presenting with multiple types of arrhythmias, in particular those accompanied by LV structural abnormalities of unknown causes.

## Limitations

A comprehensive family tree of this proband was not obtained. Her parents died of other diseases than cardiac, and she had no siblings. Her son is 27 years old and was described as healthy, but she refused to provide any further information or accept any further investigation.

## Data Availability

The original contributions presented in the study are included in the article/Supplementary Material, further inquiries can be directed to the corresponding author.
